# Assessing the Reliability of Miller’s Classification and Cairo’s Classification in Classifying Gingival Recession Defects: A Comparison Study

**DOI:** 10.3390/medicina60020205

**Published:** 2024-01-25

**Authors:** Hammam I. Fageeh, Hytham N. Fageeh, Ashok Kumar Bhati, Abdulaziz Yahay Thubab, Haitham Mohammed Hassan Sharrahi, Yahya Sulaiman Aljabri, Faisal Ibrahim Alotaibi

**Affiliations:** 1 Department of Preventive Dental Sciences, College of Dentistry, Jazan University, Jazan 45142, Saudi Arabia; hfageeh@jazanu.edu.sa; 2 College of Dentistry, Jazan University, Jazan 45142, Saudi Arabia; azoozthobab2018@gmail.com (A.Y.T.); haitham.shrahizz@gmail.com (H.M.H.S.); yahya.2000.sulaiman@gmail.com (Y.S.A.); feisal1419@gmail.com (F.I.A.)

**Keywords:** gingival recession, Miller’s classification, Cairo’s classification, classification, gingival treatment, reliability, agreement

## Abstract

*Background and Objectives:* Gingival recession results in adverse aesthetics and root sensitivity, and there is a need to treat and prevent its further progression. To overcome these problems, various advances have been made by clinicians in treating gingival recession based on the type of gingival recession. Miller’s classification has been used for a long time to classify the type of recession. However, certain limitations have been found with use of Miller’s classification such as a lack of clarity in the method for measuring soft and hard tissue loss in the interproximal area. Cairo classification was proposed to overcome limitations of Miller’s classification to classify gingival recession. Cairo’s classification is a treatment-oriented classification based on buccal and interproximal attachment loss. Therefore, the study was conducted to assess and compare the reliability of Miller’s and Cairo’s classifications in determining gingival recession. *Material and methods:* A total of 220 buccal gingival recession defects were included in the study based on the inclusion and exclusion criteria. Four examiners were included in the study. Two examiners classified the recession defects according to Miller’s classification and the other two examiners classified recession defects according to Cairo’s classification at baseline and at a 1-week interval. Statistical analysis was conducted using SPSS software version 25.0 using Cohen’s kappa correlation coefficient and Chi-square test statistics to determine the intra- and inter-rater agreement among the examiners for the two gingival recession classification systems. A *p* value of <0.05 was considered statistically significant. *Results:* The intra-rater agreement for Cairo’s classification was 0.86 and 0.82, whereas for Miller’s classification, it was found to be 0.68. The inter-rater reliability agreement for Cairo’s classification was 0.82 and 0.8, whereas for the Miller’s classification, it was 0.56 and 0.67. *Conclusions:* Within the limitations of the study, it was found that Cairo’s classification is clearer and more reliable than Miller’s classification in the assessment of gingival recession.

## 1. Introduction

Gingival recession is defined as the apical shift of the gingival margin below the cementoenamel junction (CEJ) (American Academy of Periodontology 1996) [1], resulting in root exposure and attachment loss. Therefore, gingival recession is associated with the destruction of both soft and hard tissue [2] and is a frequent problem seen in the population [3,4].

Based on the survey studies, 30% to 100% of people are affected by gingival recession [5,6,7,8,9,10,11]. Studies reports that with an increase in age there is an increase in prevalence and severity of gingival recession [5,8]. Albandar and Kingman reported 58% prevalence with recession ≥1 mm in ≥30 years of age with an average of 22.3% teeth per person [5].

For prevention and management of gingival recession, it is very important to understand the etiological factors causing recession. Gingival recessions have multifactorial causes. Biofilm-induced inflammation and improper tooth brushing leading to mechanical trauma are two of the most important etiological factors for gingival recession [7,10,12].

Various susceptibility factors and modifiable conditions are also associated with gingival recession [13]. The significant susceptibility factors included are thin biotype gingival tissue, absence or a narrow band (<2 mm) of keratinized tissue, probing pocket depths extending beyond the mucogingival junction and history of progressive gingival recession and/or inflammatory periodontal disease. The modifiable factors include plaque biofilm accumulation, inflammatory periodontal disease, aberrant frenal position, soft tissue clefts/deformities, traumatic oral hygiene habits, shallow vestibular depth, orthodontic tooth movement, subgingival restoration margins, smoking and systemic conditions such as diabetes [13]. Hence, the clinicians should identify these factors and modify them correspondingly. Additionally, the clinicians should have efficacious management and preventive measures to improve patient awareness about gingival recession [14].

Gingival recession needs treatment because it results in adverse aesthetics, root sensitivity and further progression [15]. To overcome these problems, various advances have been made by clinicians in the treatment of gingival recession [16,17,18,19]. The surgical techniques ranges from pedicle flap procedures (coronally positioned flaps or rotational flaps) alone or in combination with free gingival or connective tissue graft procedures (CTG), use of resorbable and non-resorbable membranes according to the principles of guided tissue regeneration (GTR) [20].

Considering the tissue trauma in harvesting soft tissue grafts, Acellular dermal matrices (ADM) from human and porcine origin and collagen matrices (CM) of porcine origin are possible substitutes for the CTG. Use of enamel matrix derivative (EMD) in conjunction with root coverage procedures is also an alternative treatment option [14].

Complete root coverage (CRC) is the ultimate goal of surgical therapy; however, the amount of root coverage also depends on various factors. Different clinical parameters involved in gingival recession should be evaluated for determining the predictable root coverage outcome [21].

Miller (1985) postulated complete root coverage (CRC) for Class I and II, partial coverage for Class III and no root coverage for Class IV [18]. Other possible prognostic factors such as baseline recession depth, interdental papilla, tooth type and interdental attachment loss [22], the interdental papilla [23] and the tooth type [24] are also likely to influence the final outcomes. On the other hand, the possible loss of interproximal attachment may also be able to predict the treatment outcome. There is a lack of agreement between practitioners regarding the type of classification best suited to the classification of gingival recession [25].

Miller’s classification has been commonly used worldwide because it is simple to use; however, a need for a more comprehensive classification was raised due to certain problems seen with this classification [21]. The Miller’s classification is categorized into four types depending on the relation of the gingival margin to the mucogingival junction (MGJ) and soft/hard tissue loss in the interproximal area [18]. In Miller’s Class I and Class II there is no interdental tissue loss. In Class I, the gingival margin is away from MGJ, but in Class II, the recession gingival margin may or may not extend to MGJ. It was reported that differentiating between Miller’s Class I and II was difficult. In Miller’s classification, the mucogingival junction is used as a main criterion, but the method of detection of MGJ is not described in the classification, whether using a probe or using a colored solution. The difficulty in identifying the MGJ creates confusions in identification between Class I and II. Moreover, Miller’s classification cannot be used to classify recession in the palatal surface of maxillary teeth due to the lack of MGJ in the palatal surface [25].

Additionally, the measurement of hard/soft tissue loss in the interproximal area is used to differentiate between Class III and IV in Miller’s classification [21]. In Class III, there is slight interdental tissue loss, but in Class IV, the interdental tissue loss is severe. However, the method of determining the tissue loss is not clearly defined in Miller’s classification as it is clearly distinct in Cairo’s classification by measurement of interproximal attachment loss [25].

Considering the limitations with Miller’s classification, new classification systems for gingival recession were suggested. In 2010, Mahajan et al. suggested the need for a new classification system to diagnose recession and improve the uniformity of the diagnosis and the standardization of clinical cases [26].

In 2011, Cairo et al. conceptualized a treatment-oriented classification based on buccal and interproximal attachment loss [21]. Cairo et al. (2011) explained the grey areas between the Miller classes and reorganized and simplified them based on the interproximal attachment level [21]. Cairo’s recession type 1 (RT1) was combined with Miller’s Class I and Class II, as both have favorable anticipated treatment results. Recession type 2 (RT2) and recession type 3 (RT3) point to a clear-cut differentiation between Miller’s Classes III and IV.

In Cairo’s classification, the assessment of the level of interproximal attachment loss in relation to mid buccal attachment loss is used. If the interproximal attachment loss is above or equal to the mid buccal attachment level, the recession is classified as RT2, whereas if the interproximal attachment loss is more than the mid buccal attachment loss, then it is categorized as RT3.

Although the drawbacks of Miller’s classification are highlighted and Cairo’s classification is gaining popularity because it is more objective in assessment, there is still a lack of studies comparing the accuracy of gingival recession assessment using these two classification systems. Therefore, the aim of the study was to assess and compare Miller’s and Cairo’s gingival recession classification systems and analyze the findings based on inter- and intra-rater agreements.

## 2. Material and Methods

### 2.1. Ethical Approval

The ethical approval for the study was obtained from the Standing Committee for Scientific Research, Jazan University (reference no. REC-44/05/397). Informed consent was obtained from all patients to use the collected data in the described context.

### 2.2. Study Population

Patients visiting the College of Dentistry, Jazan University, from December 2022 to March 2023 were included in the study. The patients were examined for the presence of gingival recession defects. The following exclusion and inclusion criteria were followed in the study.

#### 2.2.1. Inclusion Criteria

1. Patients with single or multiple buccal gingival recession.
2. Detectable cemento-enamel junction (CEJ) at tooth with gingival recession.
3. Patients with good oral hygiene with Sillness and plaque score less than 1.
4. Patients with bleeding on probing less than 10%.

#### 2.2.2. Exclusion Criteria

1. Patients without buccal gingival recession.
2. Patients with a plaque score greater than 1.
3. Patients with bleeding on probing greater than 10%.
4. Tooth with a prosthetic crown or restoration involving CEJ.
5. Presence of dental/root abrasion at the CEJ level.

A total of 98 patients were examined in the study. In total, 33 patients were excluded due to absence of gingival recession, 14 patients were excluded due to plaque score greater than 1, and 17 patients had bleeding score greater than 10%. Only 34 patients met the inclusion criteria.

In total, 252 buccal gingival recession defects were examined in 34 patients. A total of 8 recession defects were excluded due to restoration/prosthesis involving CEJ, 24 recession defects were excluded due to root abrasion, and 6 defects had undetectable CEJ. In total, 220 recession defects met the inclusion and exclusion criteria and were included in the study. The recession defects included both maxillary and mandibular arch and anterior and posterior teeth recession.

#### 2.2.3. Sample Size Estimation

Sample size was estimated using Sample Size Calculator by Wan Nor Arifin at 95% level of significance with 10% margin of error to achieve 80% power of study, assuming expected kappa coefficient of 0.66 (based on previous research by Mahajan et al., 2019 [27]). The minimum required sample size was calculated to be 217. So, we included 220 samples in the study.

### 2.3. Assessment of Gingival Recession Using Miller’s and Cairo’s Classification

Gingival recession defects were assessed by four examiners. All of the examiners were trained on Miller’s and Cairo’s gingival recession classification by American Board-Certified Periodontist with 8 years of experience. Selected defects were classified according to Miller’s classification (M) by two examiners (A.Y.T. and F.I.A) and Cairo’s classification (C) by two other examiners (H.M.H.S and Y.S.A) using a UNC-15 periodontal probe (Hu friedy, Chicago, IL, USA).

Miller (1985) proposed a gingival recession classification system based on the location of the gingival margin in relation to the mucogingival junction (MGJ) and the hard/soft tissue loss in interdental area as given in Table 1.

Cairo’s classification [21] was based on clinical attachment loss as an identifying factor, as given in Table 2.

Two different examiners (1 and 2) examined the recession defects and classified them according to Miller’s system, whereas the other two examiners (3 and 4) classified the same recession defects according to the Cairo classification system. The examiners were blinded to each other’s classifications. After 1 week, the recession defects were examined again and classified by the same examiners. No surgical treatment of the gingival recession was carried out in the study. However, the patients were informed of the recession defects and treatment methods for treating gingival recession.

The intra-rater reliability of the examiners was conducted by examining the grading of the recession for the two methods again at two different times. The inter-rater reliability was assessed within the same group by comparing the observations made using Miller’s and Cairo’s grading systems between two examiners. The inter-group reliability was assessed by comparing the observations between the Cairo’s and Millers’s groups.

The level of agreement was determined according to Landis and Koch 1977 [28].

- Poor agreement: 0.00.
- Slight agreement: 0.00–0.20.
- Fair agreement: 0.21–0.40.
- Moderate agreement: 0.41–0.60.
- Substantial agreement: 0.61–0.80.
- Almost perfect agreement: 0.81–1.00.

Statistical analysis was carried out using SPSS software version 25.0 using Cohen’s kappa correlation coefficient and Chi-square test statistics to determine the intra- and inter-agreement among the examiners for the two gingival recession classification systems. *p* value < 0.05 was considered statistically significant.

## 3. Results

A total of 220 gingival recession defects were included in the study. In total, 98 recession defects (44.55%) were in the maxillary arch, and 122 (55.45%) recession defects were in the mandibular arch. A greater number of recession defects was seen in the anterior teeth (52.27%) than in the posterior teeth (47.73%). The distribution of recession defects is shown in Table 3.

Recession defects were classified according to Miller’s [18] and Cairo’s [21] systems by different examiners. The distribution of recession defects according to Miller’s and Cairo’s classification by examiners for both of the observations is shown in Table 4 and Table 5.

The level of agreement was obtained using Landis and Koch’s (1977) criteria [27]. Table 6 and Table 7 show the intra-examiner reliability of the examiners with respect to Miller’s and Cairo’s classifications, while Table 8 and Table 9 show the inter-examiner reliability. Table 10 shows the comparison of the level of agreement between the Cairo and Miller classification systems.

Reliability was seen for both classifications; however, greater reliability was seen in the Cairo group for both the inter- and intra-rater examinations. According to Landis and Koch, the results showed almost perfect inter-rater and intra-rater agreement with the use of Cairo’s classification, whereas substantial intra- and inter-rater agreement was found with the use of Miller’s classification.

Using Miller’s classification, examiner 1, on first observation, classified 121 subjects as having Class I gingival recession. However, on the second observation, 117 subjects were classified as having Class I gingival recession. Of these 117 subjects, 105 were classified as Class I on the first observation, whereas 5 were previously classified as Class II and 7 were classified as Class III in the first observation. Additionally, at the second observation, 5 and 11 subjects who were classified as Class I at first observation were now classified as Class II and III, respectively.

## 4. Discussion

This study aimed to determine the reliability of Cairo’s classification and Miller’s classification in classifying gingival recession defects. CAL is used widely to determine the periodontal destruction or improvement in different periodontal conditions [29]. Therefore, interdental attachment loss could be used as a reliable parameter to indirectly determine bone loss [30].

In our study, we found greater intra- and inter-reliability using Cairo’s classification. This could be due to the use of CAL as identifying criteria in this approach, which is not the case for Miller’s classification, which is more subjective. The intra-rater agreement was almost perfect with kappa values from 0.82 to 0.86, with substantial to almost perfect values of 0.8 to 0.82 for the inter-rater agreement for the Cairo classification. This is in accordance with Cairo (2011) [21], who also found almost perfect inter-rater agreement. Sarlati et al. (2019) also demonstrated almost perfect inter-rater agreement using the Cairo classification [31].

However, Sarlati et al. (2019) also found almost perfect agreement using Miller’s classification. This is contrast to our study, where we found moderate inter-rater agreement and substantial to moderate intra-rater agreement [31]. The results of our study were also in contrast to Bert et al. (2015), who demonstrated almost perfect agreement with Miller’s classification in terms of inter- and intra-agreement (0.72–0.73 and 0.73–0.95). The difference in these results could be due to differences in the method of gingival recession assessment. Bert et al. assessed gingival recession defects using photographs, whereas we assessed them using the patients [32].

However, our results are in accordance with those of Mahajan et al., who also demonstrated less reliable agreement using Miller’s classification (κ = 0.57–0.68) and inter-rater observations (κ = 0.66). This could be due to the fact that Mahajan also assessed recession defects on the patients and not through assessment using photographs, as done byBert et al. [32].

Rotundo et al. (2011) also evaluated a new classification system to classify gingival recession and its level of agreement among different groups [33]. Rotundo et al. (2011) evaluated 120 gingival recession defects. The intra-rater agreement ranged from substantial to almost perfect (0.70 to 0.92) with interproximal attachment loss. Our study’s intra-rater agreement was also almost perfect (0.82–0.86); however, the inter-rater agreement was from 0.80–0.82 in our study. This difference could be due to differences in the sample size and in the parameters evaluated. Rotundo et al. assessed non-carious cervical lesions and inter-proximal attachment loss. This could have complicated the correct detection of the cementoenamel junction (CEJ), resulting in complicating the assessment of attachment loss.

In the present study, we found that there was confusion in identifying Miller’s Class I and Class II. Many of the recession defects classified as Class I in the first observation were classified as Class II in the second observation. Similarly, recession defects classified as Class II defects in the first observation were classified as Class I in the second observation. This discrepancy could be due to the discrepancy in detecting the MGJ by the examiners, as Miller’s classification does not describe the mode of detecting the MGJ. Furthermore, in Miller’s classification, it is difficult to classify a recession defect into Class III or Class I if the defect has interproximal soft/hard tissue loss, but recession does not extend to MGJ [25]. This could be the reason for classifying some recession defects as Class I recession defects in the first observation and as Class III in the second observation, and similarly, Class III recession defects classified in the first observation were classified as Class I in the second observation.

In the present study, there was discrepancy among the examiners in classifying Miller’s Class III and Class IV. Some recession defects classified as Class III in the first observation were classified as Class IV in the second observation, and similarly, some recession defects classified as Class IV in the first observation were classified as Class III in the second observation. This discrepancy was due to a lack of distinct criteria to measure the soft/hard tissue loss in the interproximal area differentiating between Miller’s Class III and Class IV, which is very clear in Cairo’s classification. Furthermore, regarding the use of mispositioning of teeth in Miller’s classification to determine between Class III and Class IV is also confusing. It is not clear in Miller’s classification what degree of malposition is to be used for Class III and Class IV [25]. Mahajan et al. 2021 also reported similar confusion between Class I and Class II and also between Class III and Class IV recession defects [26].

Gingival recession is a common concern among patients. Different surgical procedures are available for treating gingival recession to improve the aesthetics of the patient [34]. However, the classification of recession defects is an important aspect in selecting the most appropriate treatment procedure [35]. Miller’s classification seems simple, but it is not simple when taken into consideration [25].

Researchers highlighted the difficulty in diagnosing recession in a particular class using this classification type [25,26]. The present study also found similar findings. Considering the limitations of Miller’s classification and the use of buccal/interproximal attachment loss as identifying criterion in Cairo’s classification, Cairo’s classification should be used in clinical practice to classify gingival recession.

## 5. Conclusions

Within the limitations of the study, it was found that Cairo’s classification for classifying gingival recession defects is clearer and more reliable than Miller’s classification as it uses clinical attachment loss as an identifying criterion. However, further studies with surgical intervention for treating recession defects should be conducted to compare the root coverage outcomes for Miller’s classification and Cairo’s classification.

## Figures and Tables

**Table 1:** Miller’s classification of gingival recession [18].

Miller’s Class	Criteria
Class I	Marginal tissue recession does not extend to the mucogingival junction. There is no loss of bone or soft tissue in the interdental area.
Class II	Marginal tissue recession extends to or beyond the mucogingival junction. There is no loss of bone or soft tissue in the interdental area.
Class III	Marginal tissue recession extends to or beyond the mucogingival junction. There is bone and soft tissue loss interdentally or mispositioning of the tooth.
Class IV	Marginal tissue recession extends to or beyond the mucogingival junction. There is severe bone and soft tissue loss interdentally or severe tooth malposition.

**Table 2:** Cairo’s classification of gingival recession [21].

Cairo’s Class	Criteria
RT 1	Gingival recession with no loss of interproximal attachment. Interproximal CEJ is clinically not detectable at both mesial and distal aspects of the tooth.
RT 2	Gingival recession associated with loss of interproximal attachment. The amount of interproximal attachment loss (measured from the interproximal CEJ to the depth of the interproximal sulcus/pocket) is less than or equal to the buccal attachment loss (measured from the buccal CEJ to the apical end of the buccal sulcus/pocket).
RT 3	Gingival recession associated with loss of interproximal attachment. The amount of interproximal attachment loss (measured from the interproximal CEJ to the apical end of the sulcus/pocket) is greater than the buccal attachment loss (measured from the buccal CEJ to the apical end of the buccal sulcus/pocket).

**Table 3:** Distribution of gingival recession defects.

Arch	Number of Recession Defects		
	Anterior Teeth	Posterior Teeth	Total
Maxillary	51	47	98
Mandibular	64	58	122
Total	115	105	220

**Table 4:** Distribution of gingival recession defects according to Miller’s classification.

Miller’s Classification		Class I	Class II	Class III	Class IV
Examiner 1	1st Observation	121	10	75	14
	2nd Observation	117	11	82	10
Examiner 2	1st Observation	137	11	51	21
	2nd Observation	123	12	72	13

**Table 5:** Distribution of gingival recession defects according to Cairo’s classification.

Cairo’s Classification		RT1	RT2	RT3
Examiner 3	1st Observation	121	80	19
	2nd Observation	119	87	14
Examiner 4	1st Observation	124	77	19
	2nd Observation	128	76	16

**Table 6:** Intra-examiner reliability of examiners 1 and 2 with respect to Miller’s classification.

	Miller’s Classification		2nd Reading				Total	Kappa Correlation Coefficient	*p*-Value
			Class I	Class II	Class III	Class IV			
Examiner 1	1st reading	Class I	105	5	11	0	121	0.684	0.043 *
		Class II	5	5	0	0	10		
		Class III	7	0	64	4	75		
		Class IV	0	1	7	6	14		
	Total		117	11	82	10	220		
Examiner 2	1st reading	Class I	117	5	15	0	137	0.688	0.043 *
		Class II	5	6	0	0	11		
		Class III	1	1	47	2	51		
		Class IV	0	0	10	11	21		
	Total		123	12	72	13	220		

* *p*-value < 0.05 was considered statistically significant.

**Table 7:** Intra-examiner reliability of examiners 3 and 4 with respect to Cairo’s classification.

	Cairo’s Classification		2nd Reading			Total	Kappa Correlation Coefficient	*p*-Value
			RT1	RT2	RT3			
Examiner 3	1st reading	RT1	115	6	0	121	0.860	0.036 *
		RT2	4	75	1	80		
		RT3	0	6	13	19		
	Total		119	87	14	220		
Examiner 4	1st reading	RT1	119	5	0	124	0.825	0.036 *
		RT2	9	66	2	77		
		RT3	0	5	14	19		
	Total		128	76	16	220		

* *p*-value < 0.05 was considered statistically significant.

**Table 8:** Inter-examiner reliability of examiners 1 and 2 with respect to Miller’s classification.

	Miller’s Classification		Examiner 2				Total	Kappa Correlation Coefficient	*p*-Value
			Class I	Class II	Class III	Class IV			
1st reading	Examiner 1	Class I	109	4	6	2	121	0.569	0.048 *
		Class II	3	5	2	0	10		
		Class III	25	1	41	8	75		
		Class IV	0	1	2	11	14		
	Total		137	11	51	21	220		
2nd reading	Examiner 1	Class I	107	4	6	0	117	0.676	0.043 *
		Class II	5	5	1	0	11		
		Class III	11	3	61	7	82		
		Class IV	0	0	4	6	10		
	Total		123	12	72	13	220		

* *p*-value < 0.05 was considered statistically significant.

**Table 9:** Inter-examiner reliability of examiners 3 and 4 with respect to Cairo’s classification.

	Cairo’s Classification		Examiner 4			Total	Kappa Correlation Coefficient	*p*-Value
			Class I	Class II	Class III			
1st reading	Examiner 3	Class I	116	5	0	121	0.820	0.036 *
		Class II	7	68	5	80		
		Class III	1	4	14	19		
	Total		124	77	19	220		
2nd reading	Examiner 3	Class I	116	3	0	119	0.80	0.037 *
		Class II	11	70	6	87		
		Class III	1	3	10	14		
	Total		128	76	16	220		

* *p*-value < 0.05 was considered statistically significant.

**Table 10:** Level of agreement in the use of the classification systems for gingival recession.

		Cairo Classification		Miller’s Classification	
		Cohen’s Kappa Coefficient	*p*-Value	Cohen’s Kappa Coefficient	*p*-Value
Intra-rater	Examiner 1	0.860	0.036 *	0.684	0.043 *
	Examiner 2	0.825	0.036 *	0.688	0.043 *
Inter-rater	Observation 1	0.820	0.036 *	0.569	0.048 *
	Observation 2	0.80	0.037 *	0.676	0.043 *

* *p*-value < 0.05 was considered statistically significant.

## Data Availability

Data are included in the study.
